# A Recyclable, Adhesive, and Self-Healing Ionogel Based on Zinc–Halogen Coordination Anion Crosslinked Poly(ionic Liquid)/Ionic Liquid Networks for High-Performance Microwave Absorption

**DOI:** 10.3390/gels11060436

**Published:** 2025-06-05

**Authors:** Lei Wang, Jie Liu, Meng Zong, Yi Liu, Jianfeng Zhu

**Affiliations:** 1 Shaanxi Key Laboratory of Green Preparation and Functionalization for Inorganic Materials, School of Materials Science and Engineering, Shaanxi University of Science and Technology, Xi’an 710021, China; 18391381072@163.com (J.L.); liuyi@sust.edu.cn (Y.L.); zhujf@sust.edu.cn (J.Z.); 2 The MOE Key Laboratory of Material Physics and Chemistry under Extraordinary Conditions, Ministry of Education, School of Chemistry and Chemical Engineering, Northwestern Polytechnical University, Xi’an 710129, China

**Keywords:** poly(ionic liquids), microwave absorber, recycle, self-healing ability

## Abstract

In the past, powder-like microwave absorbers have made notable breakthroughs in performance enhancements, but complicated processes and undesirable properties have limited their practical application. Herein, a novel poly(ionic liquid) (PIL)-based ionic gel with excellent microwave absorption properties was prepared via a facile UV-initiated polymerization method. By simply adjusting the mole ratio of the polymerizable ionic liquid (IL)monomer and the IL dispersion medium, the microwave absorption properties of the obtained ionic gels can be tuned. A maximum reflection loss (RL_max_) of −45.7 dB and an effective absorption bandwidth (EAB) of 8.08 GHz were achieved, which was mainly ascribed to high ionic conduction loss induced by the high content of the dispersion medium. Furthermore, it displayed recyclable, adhesive, and self-healing properties, thus providing a new candidate for developing efficient microwave absorbers for practical applications.

## 1. Introduction

The wide application of smart wearable devices in the Internet of Things, environment detection, and healthcare has greatly improved the quality of people’s lives [1]. However, the electronic chips packaged in wearable devices inevitably produce copious amounts of electromagnetic (EM) radiation; the cumulative effects of this radiation exposure are becoming increasingly prominent with prolonged device usage time and increasing wearing density, which unavoidably poses a threat to human health [2]. Microwave absorbing materials are specialized functional materials that can effectively absorb and attenuate electromagnetic wave energy through converting it into heat, which are usually used to address the above EM interference issues. Particularly in wearable application scenarios, microwave absorbing materials should possess not only excellent microwave absorption performance but also flexible, stretchable, and adhesive properties.

To meet all the requirements for practical application, significant efforts have been devoted to developing high-performance EM absorbers, including graphene [3], Mxenes [4], and MoS_2_ [5], and great progress has been achieved. These novel nanomaterials demonstrate exceptional electromagnetic wave dissipation characteristics due to their unique dielectric properties and tunable microstructures. However, the present research is mostly focused on powder-like microwave absorbers, which must be filled into a matrix to form a slab absorber for further utilization in most cases, and the complicated processing procedure for the uniform dispersion should be conducted to obtain excellent microwave absorption, greatly hindering their further applications. The above processing limitation is particularly evident in emerging use cases such as flexible electronics and wearable devices. Recently, polymer gels composed of three-dimensional cross-linked polymer networks and large amounts of dispersion media (water, organic solvent, ionic liquid, etc.) have garnered a great deal of attention in microwave absorbing research due to the following advantages: (a) Gel materials can be prepared by simply polymerizing the monomer solution, resulting in a perfectly homogeneous dispersion of media within the polymer network [6]; (b) The dispersion media are typically polar liquids, which are capable of producing strong polarization loss to dissipate EM wave energy, allowing the corresponding microwave absorption properties to be adjusted based on the medium’s content [7]; (c) Gel materials usually display stretchable, self-healing, and adhesive properties due to the presence of noncovalent interactions in the network [8,9], which is conducive to further practical applications. Among gel materials, ionogels inherit the merits of ionic liquids (ILs); they possess not only nonvolatility and nonflammability but also high chemical stability and ionic conductivity, indicating their potential as microwave absorbers. Wu et al. [10] prepared a polyacrylamide hydrogel, polyacrylamide-DMSO organogels, and poly(acrylamide-co-acrylic acid)-poly(acrylamide-co-acrylic acid) ionogel via one-step radical polymerization and compared all of their microwave absorption properties. The results showed that the ionogel achieved wider effective absorption bandwidths (EAB) of 5.59 GHz at a matching thickness of 2.2 mm. However, due to the restricted compatibility of ILs and traditional polymer skeletons, such as poly(acrylamide), poly(acrylic acid), and poly(vinyl alcohol), the ILs did not firmly integrate with the polymeric supporting matrix and were threatened by leakage when encountering mechanical stress during application [11]. This critical limitation significantly compromises their long-term reliability in dynamic environments such as flexible electronics. Compared with traditional polymers, poly(ionic liquid)s (PILs), composed of only IL repeating units, possess similar physicochemical properties to IL, and developing ionogels by combining PILs with ILs sharing similar ionic structures would effectively address the leakage issues associated with traditional ionogels. Furthermore, the PILs could improve the ionic conductivity of the obtained ionogels [12], which is beneficial for strengthening comprehensive microwave absorption. In addition to improving microwave absorption, PILs could enhance physical properties, such as adhesive characteristics, mechanical properties, and physical stability [13], which is conducive to greatly expanding their application scenarios in the electromagnetic protection field.

To further confirm the aforementioned hypothesis, imidazolium-based PILs and ILs were selected to fabricate ionogels and investigate their possible application in the microwave absorbing field due to their relatively mature synthesis technology, high ionic conductivity, and low price. In this work, imidazolium-based ionogels were successfully prepared via the UV-initiated polymerization of 1-butyl-3-vinylimidazolium bromide ([BVIm]Br) monomers in a mixture of 1-butyl-3-methylimidazole bromide ([BMIm]Br) and anhydrous ZnCl_2_. The results showed that the obtained ionogels possessed not only recyclable, adhesive, and self-healing properties but also excellent microwave absorption performance. More significantly, the microwave absorbing properties of ionogels could be tuned by simply adjusting the mole ratio of [BVIm]Br and [BMIm]Br, thus providing a feasible method to achieve efficient microwave absorption with multifunctionality in a simple manner.

## 2. Results and Discussion

**Figure 1:**
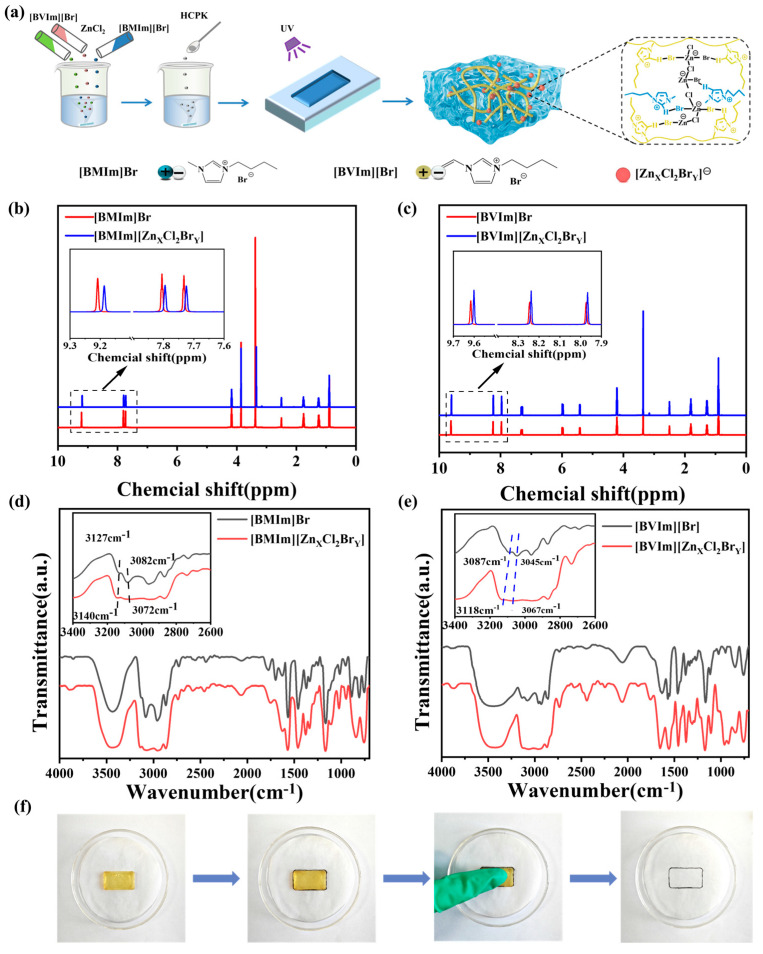
(**a**) Schematic diagram of the ionogel preparation process; (**b**) ^1^H NMR and (**d**) FT-IR spectra of [BMIm]Br and [Bmim][Zn_X_Cl_2_Br_Y_]; (**c**) ^1^H NMR and (**e**) FT-IR spectra of [BVIm]Br and [Bmim][Zn_X_Cl_2_Br_Y_]; (**f**) durability test of IG-[6].

The chemical structures within the PIL ionogels of this study are shown in Figure 1a. Zinc–halogen coordination anion crosslinked PIL ionogels were prepared via the one-pot photo-initiated polymerization of [BVIm]Br in a transparent homogeneous mixture of [BMIm]Br and anhydrous ZnCl_2_ (the molar ratio of [BVIm]Br to anhydrous ZnCl_2_ was 10:1). To tune the physical properties and microwave absorption performances of PIL ionogels, the molar ratios of [BVIm]Br and [BMIm]Br were changed to 6:4, 8:2, and 10:0, and the corresponding obtained samples were termed IG-[6], IG-[8], and IG-[10] based on the value of [BVIm]Br.

The three-dimensional structure of zinc–halogen coordination anion crosslinked PIL ionogels was characterized by ^1^H NMR and FT-IR spectra. As shown in Figure 1a,b, the presence of ZnCl_2_ caused a chemical shift in the hydrogens in the imidazolium cation of both [BMIm]Br and [BVIm]Br to the low field, indicating that the hydrogen bonding interactions between imidazolium cation and zinc–halogen coordinating anion ([Zn_x_Cl_2_Br_y_]^−^) were formed by ZnCl_2_ and the Br^−^ anion [14]. The above result was further verified by the corresponding FT-IR spectra. As shown in Figure 1c,d, a shift in the characteristic C-H stretching vibrations peak of the imidazolium ring in both [BMIm]Br and [BVIm]Br was observed, which indicated the weakening effect of Br^−^ on imidazolium cations [15,16], because of the formation of [Zn_x_Cl_2_Br_y_]^−^ anion. Therefore, the supramolecular networks within the obtained PIL ionogels are formed via interactions between the [Zn_x_Cl_2_Br_y_]^−^ anion and the imidazolium cation (Figure 1a). Due to the above supramolecular networks, [BMIm]Br can be tightly bound to the PIL chains without concern about its leakage. To test the durability of prepared IG samples, a squeezing test was conducted on the IG-[6], resulting in no leakage of [BMIm]Br on the plain paper (Figure 1f).

**Figure 2:**
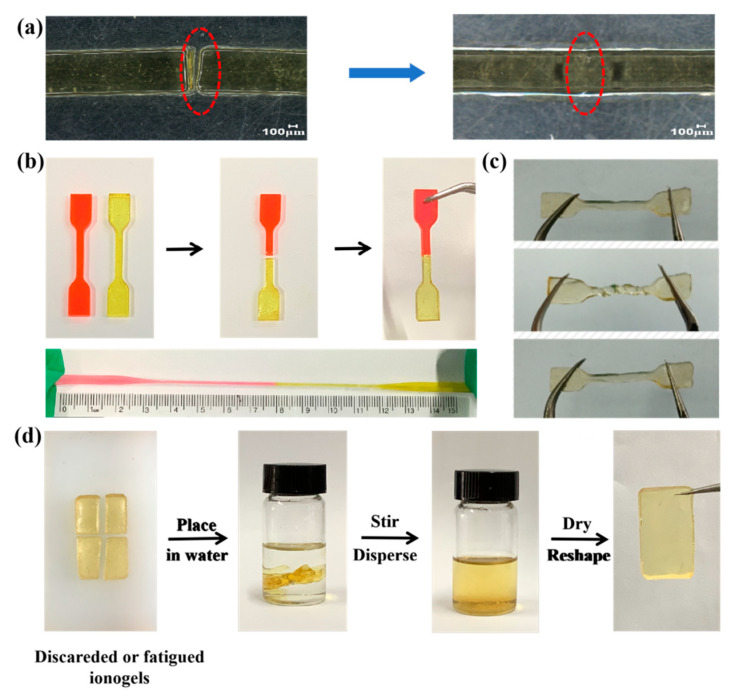
(**a**,**b**) Self-healing properties of IG-[6]; (**c**) photograph of IG-[6] being twisted at ambient temperature; (**d**) illustration of the recyclability of IG-[6].

Due to the dynamic and reversible characteristics of the hydrogen-bonding interactions between the [Zn_x_Cl_2_Br_y_]^−^ anion and the imidazolium cation, the obtained PILs displayed self-healing properties. To test the self-healing capability of IG-[6], the dumbbell-shaped sample was cut into two pieces using a razor blade (Figure 2a,b), and the corresponding self-healing process was surveyed using an optical microscope. It is clear that the two separated pieces merged into one block, and the crack disappeared after lightly making contact with each other for 20 min under conditions of 60 °C. Furthermore, the healed PILs could bear large stretching deformation without splitting, indicating their excellent self-healing capacities (Figure 2b). Apart from the self-healing character, the IG-[6] sample exhibited good elasticity at both sub-zero (−15 °C) (Figure S1) and ambient temperatures (Figure 2c) and could be twisted to recover its original flatness without any fracture, demonstrating its remarkable wide-temperature stability in practical applications. Remarkably, as shown in Figure 2d, IG-[6] can be recycled and reused via the solvent-induced dissolution method, and the fatigued ionogels can be completely dissolved in a good solvent and water and return to a gel state after removing water by heating. Due to its unique self-healing and recyclable properties, the material maintains stable performance even in complex environments, thereby significantly expanding its application scope and practical value.

**Figure 3:**
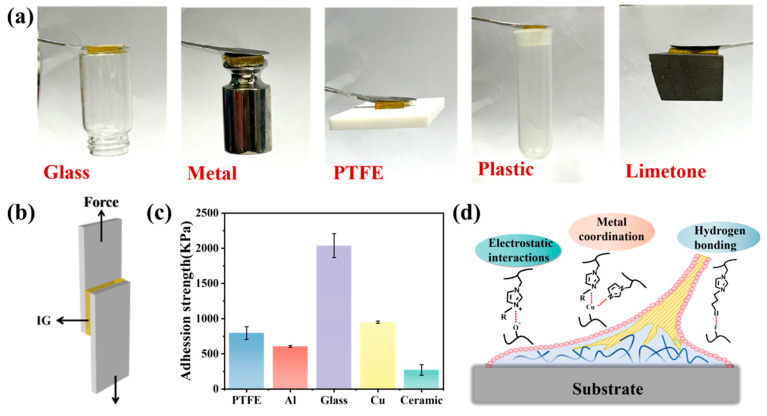
(**a**) The adhesion performance of IG-[6] on different substrates; (**b**) corresponding adhesion mechanisms of IG-[6] on PTFE, Al, glass, Cu, and Al. (**c**) The adhesion strengths and (**d**) corresponding adhesion mechanisms of IG-[6].

In the design process of composite materials, the adhesive properties of an absorbing material should be considered a key factor during the application process because they directly affect the overall performance and durability of the material. As shown in Figure 3a, the prepared IG-[6] sample exhibited good adhesive properties and was shown to adhere to various substrates, such as glass, metal, PFFE, ceramic, and limestone. The adhesion strengths of IG-[6] were quantitatively measured using lap-shear tests on various surfaces (Figure 3b); tensile adhesion strengths corresponding to PTFE, aluminum, glass, copper, and ceramic were 796, 605, 2037, 951, and 271 KPa, respectively (Figure 3c). The strong adhesion property of IG-[6] can be attributed to the interactions between the ionogels and the substrates, which involve hydrogen bonds, electrostatic interactions, and metal coordination (Figure 3d) [17,18,19].

**Figure 4:**
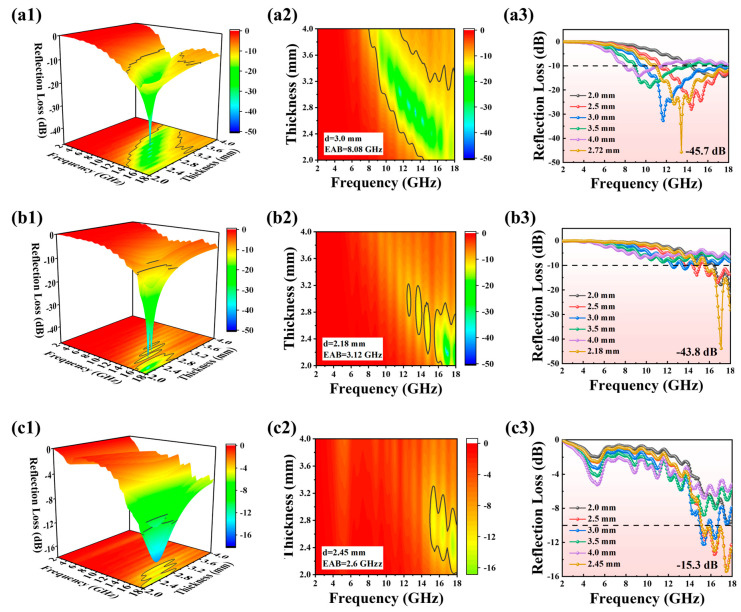
The 3D RL projection plots (1), 2D RL diagrams (2), and RL curves (3) of (**a**) IG-[6], (**b**) IG-[8], and (**c**) IG-[10] in the 2–18 GHz range.

**Figure 5:**
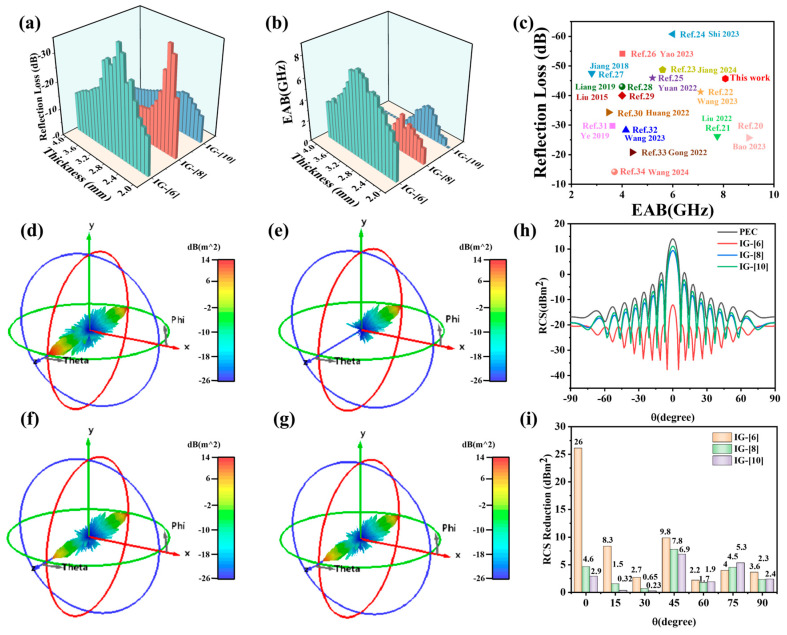
(**a**) The bar diagrams of the RL values, (**b**) the bandwidth of ionic gels at the different thicknesses, and (**c**) the comparison of RL_max_ and EAB with other EMW absorbers. Three-dimensional simulation model of (**d**) PEC, (**e**) IG-[6], (**f**) IG-[8], and (**g**) IG-[10]. (**h**) RCS value of PEC and ionic gels. (**i**) RCS reduced values of ionic gels at different angles [20,21,22,23,24,25,26,27,28,29,30,31,32,33,34].

Figure 4 shows the 3D RL projection plots, 2D RL diagrams, and RL curves for IG-[6], IG-[8], and IG-[10]. As displayed in Figure 4a, the RL_max_ and EAB of IG-[6] were −45.7 dB and 8.08 GHz (9.92–18 GHz). When the molar ratio of [BVIm]Br and [BMIm]Br was increased to 8:2 in ionogels, the microwave absorption performance of IG-[8] was worse, and both the RL_max_ and EAB values decreased notably. As shown in Figure 4b, the RL_max_ value of IG-[8] decreased to −43.8 dB, and the corresponding EAB only covered 3.12 GHz. When the molar ratio of [BVIm]Br and [BMIm]Br was further increased to 10:0, as illustrated in Figure 4c1–c3, a much remarkable decrease in the microwave absorption properties for IG-[10] was observed, and the values of RL_max_ and EAB further decreased to −15.4 dB GHz and 2.88GHz, respectively. To identify the better microwave absorption performance of IG-[6], a comparison between the RL and EAB in a matching range of thickness from 2.0 mm to 4.0 mm for the three ionogels was conducted and is shown in Figure 5a,b. It can be seen that IG-[6] displayed better comprehensive microwave absorption in the wide GHz range compared to IG-[8] and IG-[10]. To further assess the microwave absorption performance of IG-[6], a comparison of EAB and RL_max_ with other microwave absorbers was conducted and is shown in Figure 5c [20,21,22,23,24,25,26,27,28,29,30,31,32,33,34]. The detailed information on various materials is listed in Table S1. The complex microwave absorbing property of the IG-[6] is on the top layer with respectable reflection loss intensities and comparable EAB. The damage to the radar absorbing coating is inevitable in the use process, and the microwave absorbing performance after self-healing should be investigated. The microwave absorption properties of IG-[6] after self-healing were measured by cutting the concentric ring-shaped sample into two pieces and then recontacting the fresh-cut surfaces for 20 min under conditions of 60 °C. As shown in Figure S3, the RLmax and EAB after self-healing were −27.4 dB and 7.68 GHz, respectively, indicating its good absorbing stability.

To better understand the potential of ionic gels in practical applications, a radar-cross-section (RCS) simulation was carried out using CST, with the RCS value directly reflecting the material’s electromagnetic wave absorption capability; the smaller the RCS values, the better the absorption properties. Figure 5d–g show the 3D radar scattering signals of different intensities for the perfect electric conductor (PEC), PEC/IG-[6], PEC/IG-[8], and PEC/IG-[10]. For the strongest scattering signal, the smallest values for IG-[6] were observed among the above four materials, demonstrating the best potential for further practical application. Figure 5h displays the RCS value of the ionic gels at different incidence angles, and it can be observed that IG-[6] had the lowest RCS value in most incidence angles from −90° to 90°, which was lower than −10 dB m^2^. To further investigate the effect of the ionic gels coating on the reduction in the RCS values, the corresponding decreased values were obtained by subtracting the background PEC from the RCS value of the ionic gels, and the results are shown in Figure 5i. It is evident from the figure that the PILs had a significant reduction effect on RCS values, and a maximum RCS reduction of 26 dBm^2^ was achieved for IG-[6]. The above simulation results are consistent with the microwave absorption obtained in the 2–18 GHz range using the coaxial fixture, suggesting great application prospects in the electromagnetic protection field.

**Figure 6:**
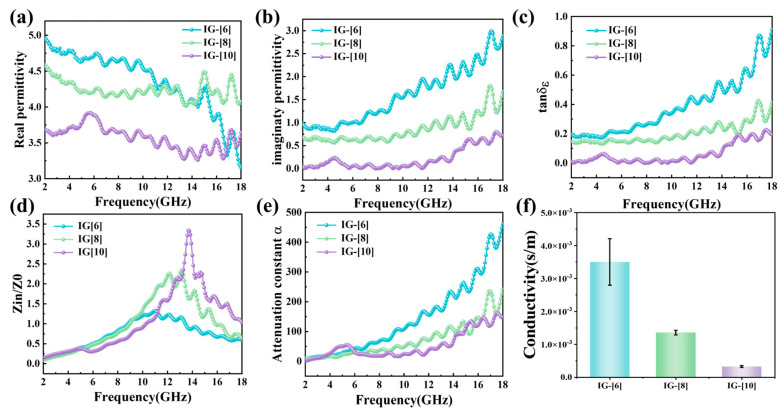
Electromagnetic characteristics of ionic gels: (**a**) ε′, (**b**) ε″, (**c**) $\tan\delta_{\varepsilon }$. (**d**) |Z_in_/Z_0_|, (**e**) attenuation constant, and (**f**) ionic conductivity plots of ionic gels.

The effect of the molar ratio of [BVIm]Br and [BMIm]Br on the final microwave absorbing properties of the ionic gels was further studied based on their electromagnetic parameters (complex permittivity (ε_r_ = ε′ − jε″) and complex permeability (μ_r_ = μ′ − jμ″)) [35], which were gauged using the coaxial method within the typical frequency range of 2–18 GHz. Since there were no magnetic groups in the ionic gels, the corresponding values of μ′ and μ″ were almost equal to 1 and 0; thus, the EM wave absorption performance of ionic gels depended only on their ε_r_. The real part (ε′) represents the material’s ability to store electrical energy under an external electric field, reflecting its polarization characteristics. A larger real-part value indicates the material’s stronger ability to store electromagnetic field energy. The imaginary part (ε″) characterizes the material’s ability to dissipate electrical energy in an alternating electric field, reflecting its dielectric loss properties. A larger imaginary part value indicates a stronger ability of the material to convert electromagnetic energy into thermal energy. As evident in Figure 6a,b, both ε′ and ε″ values showed a decreasing trend with an increase in the mole fraction of polymerizable [BVIm]Br across most frequency bands, which could be attributed to the decrease in ionic conductivity (Figure 6f) caused by an increase [BVIm]Br content in the ionic gels. Generally, the ionic conductivity of the ionic gels is inversely correlated to that of the polymer content, and a higher content of polymerizable components results in a densely packed structure and more difficult ionic migration, and thus a lower conductivity. For IG-[6], IG-[8], and IG-[10], the ε′ (and ε″) values fluctuated in the ranges of 3.1–5.0 (1.0–3.0), 4.0–4.6 (0.6–1.8), and 3.2–3.9 (0–0.8), respectively. Furthermore, multiple resonance peaks at 2–18 GHz were observed in the ε″ curves of all the ionic gels, demonstrating the presence of multiple relaxation processes originating from interfacial polarization and dipole polarization. Herein, the former would be induced by a large number of solid/liquid interfaces between the polymer framework and dispersion medium, and the latter would be associated with the N functional group and the [Zn_x_Cl_2_Br_y_]^−^ anion. The presence of the above two relaxation processes is characterized by the corresponding Cole/Cole curves [36,37], illustrated in Figure S3. Multiple semicircular features are distinctly visible across all curves, indicating that interfacial polarization and dipole polarization contributed to dielectric loss. In addition, a small tail was observed in IG-[6] and IG-[8] plots but not in IG-[10], indicating that ionic conduction loss contributed to the dielectric loss of IG-[6] and IG-[8] but not to IG-[10]. For further investigation of the EM attenuation capability of the obtained ionogels, the corresponding frequency dependences of dielectric loss tangent (tanδ_ε_) were plotted [38], as displayed in Figure 6c. It is evident that the variation trend of tanδ_ε_ values for the ionic gels was consistent with that of ionic conduction (Figure 6f), further confirming the contribution of conduction loss to the dielectric loss.

To further analyze why the obtained ionogels possessed different microwave absorbing properties, the impedance-matching condition (|Z_in_/Z_o_|) and the attenuation constant (α) were analyzed using the following equation:

(1) $$Z_{in}/Z_{o}=\sqrt{\frac{\mu_{r}}{\varepsilon_{r}}}\tanh\left(j\frac{2\pi fd}{c}\sqrt{\mu_{r}\varepsilon_{r}}\right)$$

(2) $$\alpha =\frac{\sqrt{2}\pi f}{c}\times \sqrt{(\mu \prime \prime \varepsilon \prime \prime -\mu \prime \varepsilon \prime )+\sqrt{(\mu \prime \prime \varepsilon \prime \prime -\mu \prime \varepsilon \prime {)}^{2}+(\mu \varepsilon \prime +\mu \prime \varepsilon \prime \prime {)}^{2}}}$$

The impedance-matching characteristics, which determine the penetration rate of EM waves into the interior absorber, can typically be assessed using the degree of values |Zin/Zo| drawing near to 1, and a value closer to 1 indicates superior impedance matching [39]. As shown in Figure 6d, the |Zin/Zo| values of IG-[6] were closer to 1 than those of the other ionic gels over a broader frequency range, indicating better impedance matching. Generally, high ionic conduction opposes impedance matching, which can be observed in other polymer-based gels [40,41]. However, in this manuscript, we developed ionogels by combining PILs with ILs sharing similar ionic structures, and both have an effect on the ionic conduction. However, due to the limitations of the characterization technique, it is difficult to fully clarify how the ionic conduction affects the impedance matching. New methods will be developed in subsequent studies to aid in a deeper understanding. In addition, the attenuation capability improves as the attenuation constants increase [42]. As shown in Figure 6f, the α values of the PILs increased in the order IG-[6] > IG-[8] > IG-[10], verifying that the higher, comprehensive microwave absorbing properties of IG-[6] could be ascribed to the synergic action of better impedance matching and stronger attenuation constant.

**Figure 7:**
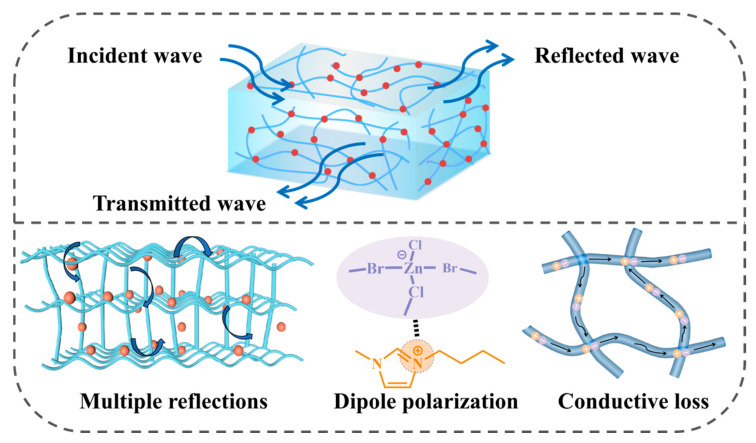
The EM absorbing mechanism of IG.

Based on the preceding analysis, an EM wave absorption mechanism for IG-[6] was proposed, as shown in Figure 7. Firstly, proper ionic conductivity endowed IG-[6] with good impedance matching, enabling most of the incident EM wave to penetrate the sample and dissipate without significant reflection. The higher ionic conduction loss derived from the higher dispersion medium content, combined with dipole polarization induced by the N heteroatom in the imidazolium ring and the [Zn_x_Cl_2_Br_y_]^−^ anion, quickly dissipated the incident EMW energy. Finally, the unique three-dimensional polymeric architecture within ionic gels promotes multiple scattering events for incoming electromagnetic radiation, thereby increasing the propagation distance and significantly enhancing energy dissipation.

## 3. Conclusions

In summary, PIL-based ionic gel microwave absorbents with excellent performance were successfully prepared by the UV-initiated polymerization of [BVIm]Br monomers in the mixture of [BMIm]Br and anhydrous ZnCl_2_. The results showed that the microwave absorption properties could be regulated by simply adjusting the mole ratio of [BVIm]Br and [BMIm]Br. The IG-[6] sample (with a [BVIm]Br and [BMIm]Br molar ratio of 6:4) demonstrated the best EM wave absorption performance. The corresponding EAB reached 8.08 GHz (9.92–18 GHz), superior to that of most reported microwave absorbers. These outstanding microwave absorbing properties can be linked to the ionic conduction loss induced by the high content of [BMIm]Br and dipole polarization originating from the N heteroatom in the imidazolium ring and the [ZnxCl2Bry]− anion. In addition to their excellent microwave absorption properties, the obtained ionic gels not only showed no risk of leakage of the dispersion medium but also displayed recyclable, adhesive, and self-healing properties. This study provides a strategy for preparing flexible microwave absorbing materials with broad application potential.

## 4. Future Directions

Future research on ionogel-based wave-absorbing materials should focus on the following key aspects: First, molecular structure design and composition optimization should be used to achieve broader EAB characteristics, which would be involved in the systematic investigation of how to select suitable ion pairs. Secondly, magnetism ionogels should be developed to enhance the low-frequency absorption properties. Finally, more accurate multiscale simulation methods should be established to reveal the structure/activity relationship between ionic structures and electromagnetic loss at the molecular level, which will provide crucial theoretical guidance for material design.

## 5. Materials and Methods

### 5.1. Materials

The materials 1-vinylimidazole and bromobutane were purchased from Meryer (Shanghai) Chemical Technology Co., Ltd. (Shanghai, China) N-methylimidazole, 1-Bromoctane, and anhydrous ZnCl_2_ were purchased from Shanghai Macklin Biochemical Technology Co., Ltd. (Shanghai, China), and 1-Hydroxycyclohexyl phenyl ketone (HCPK) was purchased from Aladdin Reagents Co., Ltd. (Shanghai, China). Methanol, diethyl ether, ethyl acetate, methyl orange, and rhodamine B were purchased from Sinopharm Chemical Reagent Co., Ltd. (Xi’an, China). Deionized water was utilized in all the experiments.

### 5.2. Methods

Synthesis of 1-Butyl-3-methylimidazole bromide ([BMIm]Br)

[BMIm]Br was synthesized by the quaternization reaction. Firstly, 18.4 g of 1-bromoalkane was added to 10.0 g of N-methylimidazole at room temperature by stirring. After stirring for 72 h, the mixture was washed with ethyl acetate and ethyl three times, yielding a colorless, transparent, oily liquid ([BMIm]Br) obtained through vacuum evaporation. ^1^H NMR (600 MHz, DMSO-*d*) δ 9.21 (s, 1H), 7.80 (s, 1H), 7.73 (s, 1H), 4.18 (s, 2H), 3.85 (s, 3H), 1.76 (s, 2H), 1.24 (s, 2H), 0.89 (s, 3H) (Figure S4).

Synthesis of 1-vinyl-3- butylimidazolium bromide ([BVIm]Br)

[BVIm]Br was synthesized using a similar method to that of [BMIm]Br. Firstly, 10.0 g of 1-vinylimidazole was added dropwise to 16.0 g of bromobutane and stirred at room temperature for 24 h. After reacting, the mixture was dissolved in methanol and washed three times with diethyl ether; the white precipitate ([BVIm]Br) was collected by filtration. 1H NMR (600 MHz, DMSO-d) δ 9.63 (s, 1H), 8.26 (s, 1H), 7.99 (s, 1H), 7.32 (s, 1H), 6.01 (s, 1H), 5.44 (s, 1H), 4.21 (s, 2H), 1.84 (s, 2H), 1.32 (s, 2H), 0.93 (s, 3H) (Figure S5).

Preparation of ionogels

Ionogels were prepared by conducting a simple one-pot photopolymerization of [BVIm]Br in a transparent and homogeneous solution of [BMIm]Br and ZnCl_2_. Firstly, [BMIm]Br, [BVIm]Br, and anhydrous ZnCl_2_ were stirred at 50 °C for 2 h to obtain a clear, transparent, and homogeneous target solution. Then, 0.5 wt% photoinitiator HCPK was added to the above solution with regard to the weight of [BVIm]Br. After stirring for 0.5 h, the above mixture was transferred to a mold, and the ionogels were prepared by irradiation with 365 nm UV light for 15 min. The obtained ionogels were labeled IG-[6], IG-[8], and IG-[10] according to the molar ratios of [BVIm]Br to [BMIm]Br, e.g., 6:4, 8:2, and 10:0, respectively.

### 5.3. Characterization

^1^H nuclear magnetic resonance (NMR) spectra of the synthesized ILs [BMIm]Br, [BVIm]Br, [BMIm][Zn_x_Cl_2_Br_y_], and [BVIm][Zn_x_Cl_2_Br_y_] were recorded using a BRUKER AVANCE NEO (600 MHz) spectrometer in DMSO-d_6_. Fourier-transform infrared (FT-IR) spectra of [BMIm]Br, [BVIm]Br, [BMIm][Zn_x_Cl_2_Br_y_], and [BVIm][Zn_x_Cl_2_Br_y_] were recorded using an IR Tracer-100 over a range of 600–4000 cm^−1^. The adhesion performance of the ionogels was evaluated by a shear test using a single column testing machine (INSTRON 5943, US) at a speed of 10 mm/min. The self-healing process of the ionogel was observed with a Keen’s depth of field three-dimensional microscope system (VHX-950F). The conductivity of synthesized ionogels was measured using an electrochemical workstation (CHI760C) based on the following equation:

$$\sigma =\frac{L}{S\times R}$$

where L is the length of the sample, S is the cross-sectional area of the sample, and R is the bulk resistance value.

To obtain the electromagnetic wave absorption properties of the ionogel, a mixture of each formulation was injected into a mold and light-cured to prepare a concentric ring-shaped gel with dimensions of 3.04 mm inner diameter, 7 mm outer diameter, and 3 mm thickness. The complex permittivity and complex permeability of the materials were measured by the coaxial method using a vector network analyzer (Agilent 5234A, Agilent Technologies, Santa Clara, CA, USA) in the frequency range of 2–18 GHz.

## Data Availability

The original contributions presented in the study are included in the article; further inquiries can be directed to the corresponding author.

## Supplementary Materials

The following supporting information can be downloaded at: https://www.mdpi.com/article/10.3390/gels11060436/s1, Figure S1. After storage under freezing conditions (−15 °C) for 24 h, twisting and rotation can be performed without deformation. Figure S2. RL curves of IG-[6] (a) before self-healing and (b) after self-healing. Figure S3. (a–c) Cole-Cole curves of IG-[6], IG-[8] and IG-[10]. Figure S4. ^1^H nuclear magnetic resonance (NMR) spectra of [Bmim]Br and [Bmim][Zn_X_Cl_2_Br_Y_] (b). Figure S5. ^1^H nuclear magnetic resonance (NMR) spectra of [Bvim]Br and [Bmim][Zn_X_Cl_2_Br_Y_]. Table S1. The value of RL_max_ and EAB for various materials in Figure 5c.

## References

[1] Wang Y.Z., Wang Y.C., Liu T.T., Zhao Q.L., Li C.S., Cao M.S. (2025). MXene Hybridized Polymer with Enhanced Electromagnetic Energy Harvest for Sensitized Microwave Actuation and Self-Powered Motion Sensing. Nano Micro Lett..

[2] Cheng Y., Li Z., Li Y., Dai S., Ji G., Zhao H., Cao J., Du Y. (2018). Rationally regulating complex dielectric parameters of mesoporous carbon hollow spheres to carry out efficient microwave absorption. Carbon.

[3] Singh S., Singh S.K., Kumar A. (2024). Influence of Reduced Graphene Oxide Flakes Addition on the Electromagnetic Wave Absorption Performance of Silicon Carbide-Based Wave Absorber. JOM.

[4] Guo Z., Zong Z., Cao Y., Zhao Y., Wang F., Luo P., Liu S., Ren F., Ren P. (2024). Hetero-structured construction of RGO nanosheets decorated by flower-like MoS_2_ toward the regulation of electromagnetic wave absorption performance. Mater. Today Phys..

[5] Aka C., Kıvrak B., Tekşen F.A. (2023). Phase (1T/2H) dependent electromagnetic wave absorbing performance of flower-like MoS_2_ nanosheet. Mater. Today Commun..

[6] Nan Z., Wei W., Lin Z., Yuan R., Ouyang J., Zhang M., Chang J., Hao Y. (2025). Arginine-inspired Ti_3_C_2_T_x_ MXenes with antioxidation function for highly air-stable electromagnetic interference shielding. Mater. Today Nano.

[7] Xu S., Wu S., Zhu R., Qiu Z., Yan Y. (2024). Fully physically crosslinked PNIPAM ionogels with high mechanical properties and temperature-managed adhesion achieved by H_2_O/ionic liquid binary solvents. Adv. Funct. Mater..

[8] Yan X., Zhao R., Lin H., Zhao Z., Song S., Wang Y. (2024). Nucleobase-driven wearable ionogel electronics for long-term human motion detection and electrophysiological signal monitoring. Adv. Funct. Mater..

[9] Zhao Z., Zhang L., Wu H. (2022). Hydro/organo/ionogels: “Controllable” electromagnetic wave absorbers. Adv. Mater..

[10] Yang H., Wu K., Zhu J., Lin Y., Ma X., Cao Z., Ma W., Gong F., Liu C., Pan J. (2024). Highly efficient and selective removal of anionic dyes from aqueous solutions using polyacrylamide/peach gum polysaccharide/attapulgite composite hydrogels with positively charged hybrid network. Int. J. Biol. Macromol..

[11] Gao J., Zeb A., Li H., Xie Y., Li Z., Zhang J., Zhang Y., Zhang S. (2024). Poly (ionic liquid) s-based ionogels for sensor applications. ACS Appl. Polym. Mater..

[12] Tavares F.C., Cholant C.M., Kohlrausch E.C. (2023). Ionic liquid boosted conductivity of biopolymer gel electrolyte. J. Electrochem. Soc..

[13] Eftelchari A., Saito T. (2017). Synthesis and properties of polymerized ionic liquids. Eur. Polym. J..

[14] Li L., Wang X., Gao S., Zheng S., Zou X., Xiong J., Li W., Yan F. (2024). High-toughness and high-strength solvent-free linear poly (ionic liquid) elastomers. Adv. Mater..

[15] Luo C., Huang Z., Guo Z.H., Yue K. (2023). Recent progresses in liquid-free soft ionic conductive elastomers. Chin. J. Chem..

[16] Luo C., Chen Y., Huang Z., Fu M., Ou W., Huang T., Yue K. (2023). A fully self-healing and highly stretchable liquid-free ionic conductive elastomer for soft ionotronics. Adv. Funct. Mater..

[17] Cedano-Serrano F.J., Sidoli U., Synytska A., Tran Y., Hourdet D., Creton C. (2019). From molecular electrostatic interactions and hydrogel architecture to macroscopic underwater adherence. Macromolecules.

[18] Saiz-Poseu J., Mancebo-Aracil J., Nador F., Busqué F., Ruiz-Molina D. (2019). The chemistry behind catechol-based adhesion. Angew. Chem. Int. Ed..

[19] Deptula A., Rangel-Galera J., Espinosa-Marzal R.M. (2023). Control of Surface Morphology, Adhesion and Friction of Colloidal Gels with Lamellar Surface Interactions. Adv. Funct. Mater..

[20] Bao S., Zhang M., Zeng Y., Li Q., Jiang Z., Xie Z. (2023). Thermal energy compensation strategy to achieve rambutan-like CoFe@ C-CNTs composites with controllable cross-linked networks for broadband microwave absorption. Carbon.

[21] Liu R., An Z., Liao B., Zhang J. (2022). FeNi alloy and nickel ferrite codoped carbon hollow microspheres for high-efficiency microwave absorption. J. Mater. Chem. C.

[22] Wang H., Qu Q., Gao J., He Y. (2023). Enhanced electromagnetic wave absorption of Fe_3_O_4_@MnO_2_@Ni–Co/C composites derived from Prussian blue analogues. Nanoscale.

[23] Jiang M., Wang S., Xu P., Shang T., Jiang Y., Liu Y., Yue X. (2024). Highly flexible hydrogels with readily adjustable electromagnetic parameter for efficient electromagnetic wave absorption. ACS Appl. Nano Mater..

[24] Shi Q., Zhao Y., Li M., Li B., Hu Z. (2023). 3D lamellar skeletal network of porous carbon derived from hull of water chestnut with excellent microwave absorption properties. J. Colloid Interface Sci..

[25] Yuan H., Zhang Y., Lu G., Chen F., Xue T., Shu X., Zhao Y., Nie J., Zhu X. (2022). Transparent organogel based on photopolymerizable magnetic cationic monomer for electromagnetic wave absorbing. J. Ind. Eng. Chem..

[26] Yao Z., Xu S., Zhang X., Yuan J., Rong C., Xiong Z., Zhu X., Yu Y., Yu H., Kang S. (2023). CuCo nanocube/N-doped carbon nanotube composites for microwave absorption. ACS Appl. Nano Mater..

[27] Jiang Y., Chen Y., Liu Y.J., Sui G.X. (2018). Lightweight spongy bone-like graphene@ SiC aerogel composites for high-performance microwave absorption. Chem. Eng. J..

[28] Liang C., Wang Z. (2019). Eggplant-derived SiC aerogels with high-performance electromagnetic wave absorption and thermal insulation properties. Chem. Eng. J..

[29] Liu X., Zhang L., Yin X., Ye F., Liu Y., Cheng L. (2015). The microstructure of SiCN ceramics and their excellent electromagnetic wave absorbing properties. Ceram Int..

[30] Huang Y., Xie Y., Zhao J., Yin X., Chai C. (2022). Variety of ZIF-8/MXene-based lightweight microwave-absorbing materials: Preparation and performances of ZnO/MXene nanocomposites. J. Phys. Chem. C.

[31] Ye X., Chen Z., Ai S., Hou B., Zhang J., Liang X., Zhou Q., Liu H., Cui S. (2019). Effects of SiC coating on microwave absorption of novel three-dimensional reticulated SiC/porous carbon foam. Ceram Int..

[32] Wang J., Tao J., Zhou J., Van Zalinge H., Yao Z., Yang L. (2023). Synthesis mechanism of different morphological SiC and its electromagnetic absorption performance. Mater. Charact..

[33] Gong G., Li R., Zhang Y., Zhang A., Liang S., Lu S., Li Z., Wang Z. (2022). Compounds, Wave-absorbing properties of Ni-Zn ferrites loaded on coal-based, densely porous light carbon functional materials. J. Alloys Compd..

[34] Wang S., Hao X., Liu Y., Cheng Z., Chen S., Peng G., Tao J., Yao J., Yang F., Zhou J. (2024). Interfaces, intelligent tunable wave-absorbing CNTs/VO_2_/ANF composite aerogels based on temperature-driving. ACS Appl. Mater. Interfaces.

[35] Ouyang J., He Z.L., Zhang Y. (2019). Trimetallic FeCoNi@C nanocomposite hollow spheres derived from metal–organic frameworks with superior electromagnetic wave absorption ability. ACS Appl. Mater. Interfaces.

[36] Ali-Zade R.A. (2016). Study of Nanocomposite Permittivity on the Basis of Magnetite Nanoparticles (Fe_3_O_4_) and Polymeric Matrix: Polyethylene and Polystyrene in Electromagnetic Field. IEEE Trans. Magn..

[37] Kuang J.L., Jiang P., Hou X., Xiao T., Zheng Q.F., Wang Q., Liu W.X., Cao W.B. (2019). Dielectric permittivity and microwave absorption properties of SiC nanowires with different lengths. Solid State Sci..

[38] Hassan A., Aslam M.A., Bilal M. (2021). Modulating dielectric loss of MoS_2_@ Ti_3_C_2_Tx nanoarchitectures for electromagnetic wave absorption with radar cross section reduction performance verified through simulations. Ceram. Int..

[39] Soman R., Kim J.M., Aiton S. (2022). Guided waves based damage localization using acoustically coupled optical fibers and a single fiber Bragg grating sensor. Measurement.

[40] Liang L., Deng L.C., Luo B.C., Zhang L.M., Tao K., Li X.X., Chen Q., Wu H.J. (2025). Flexible, large area preparable phase change PVA/P(ILs-AM)/SSD films for electromagnetic wave absorption and infrared stealth. iScience.

[41] Xie W.K., Tang Q., Xie J.L., Fei Y., Wan H.J., Zhao T., Ding T.P., Xiao X., Wen Q.Y. (2024). Organohydrogel-based transparent terahertz absorber via ionic conduction loss. Nat. Commun..

[42] Singh S.K., Akhtar M.J., Kar K.K. (2018). Hierarchical carbon nanotube-coated carbon fiber: Ultra lightweight, thin, and highly efficient microwave absorber. ACS Appl. Mater. Interfaces.

