# Supplementary material for: A Recyclable, Adhesive, and Self-Healing Ionogel Based on Zinc–Halogen Coordination Anion Crosslinked Poly(ionic Liquid)/Ionic Liquid Networks for High-Performance Microwave Absorption
Source: Gels. 2025 Jun 5;11(6):436. doi: 10.3390/gels11060436 (PMC12191830; doi:10.3390/gels11060436)
Supplement: Supplementary file 1 [file gels-11-00436-s001.zip › gels-3637991-supplementary.pdf]

Supporting information

A recyclable, adhesive and self-healable ionogels based on Zinc-halogen coordination anion crosslinked poly (ionic liquid)/ionic liquid network for high Performance microwave absorbing

Lei Wang[a]*, Jie Liu[a], Meng Zong[b]*, Jianfeng Zhua

aShaanxi Key Laboratory of Green Preparation and Functionalization for Inorganic Materials, School of Materials Science and Engineering, Shaanxi University of Science and Technology, Xi'an 710021, China

b The MOE Key Laboratory of Material Physics and Chemistry Under Extraordinary Conditions, Ministry of Education, School of Chemistry and Chemical Engineering, Northwestern Polytechnical University, Xi'an, 710129, PR China

*Email address of Correspongding autor: wangleiorganic@126.com (L. Wang),
zongmeng@nwpu.edu.cn (M. Zong)

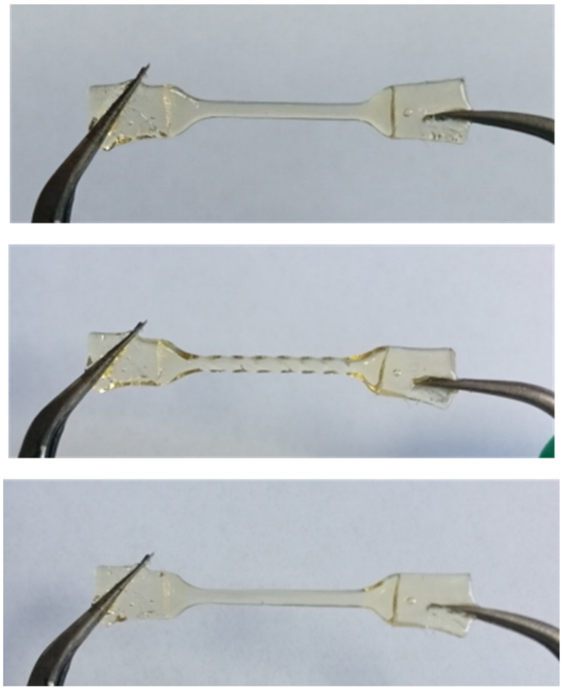

Figure S1. After storage under freezing conditions(-15℃) for 24 hours, twisting and rotation can be performed without deformation.

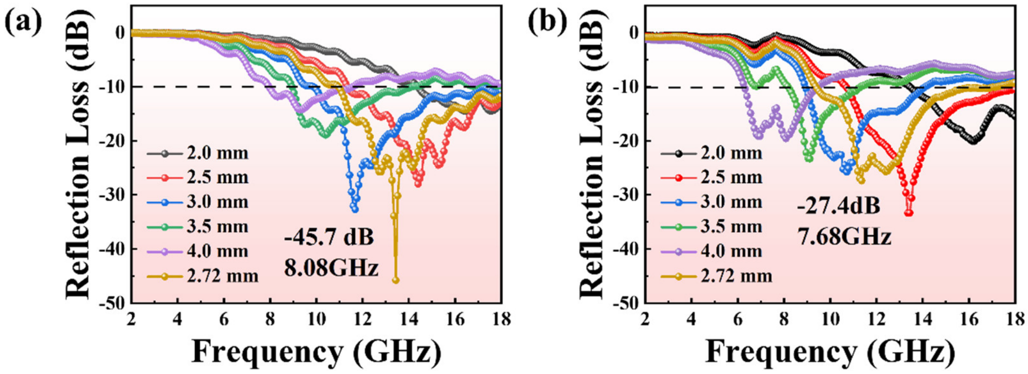

Figure S2. RL curves of IG-[6] (a) before self-healing and (b) after self-healing

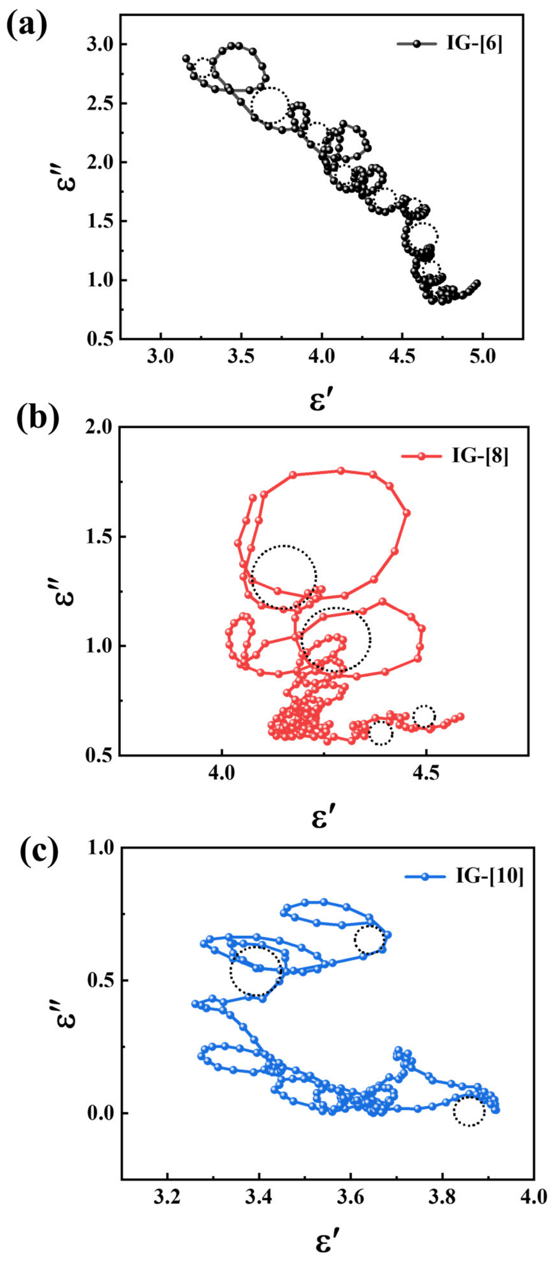

Figure S3. a-c)Cole-Cole curves of IG-[6]、IG-[8] and IG-[10]

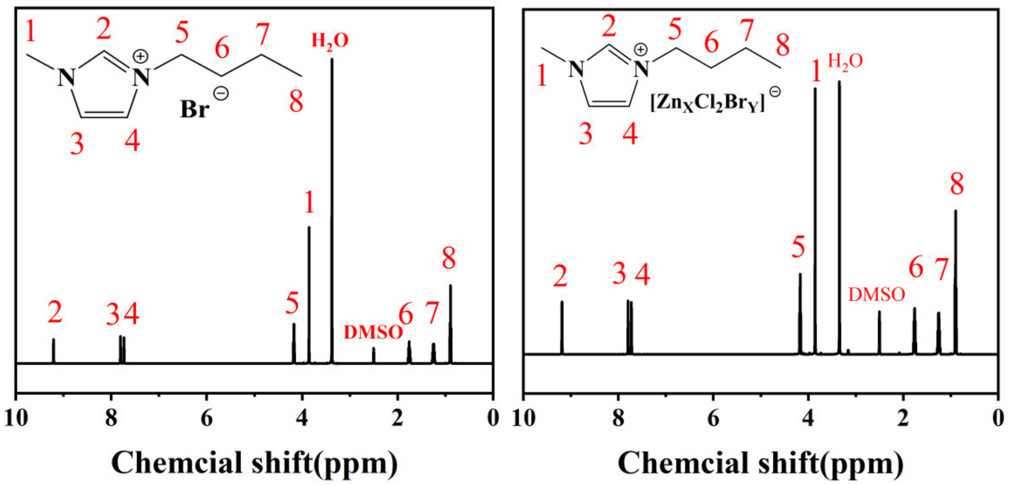

Figure S4. ^{1}H nuclear magnetic resonance (NMR) spectra of [Bmim]Br and [Bmim][$Zn_XCl_2Br_Y$] (b)

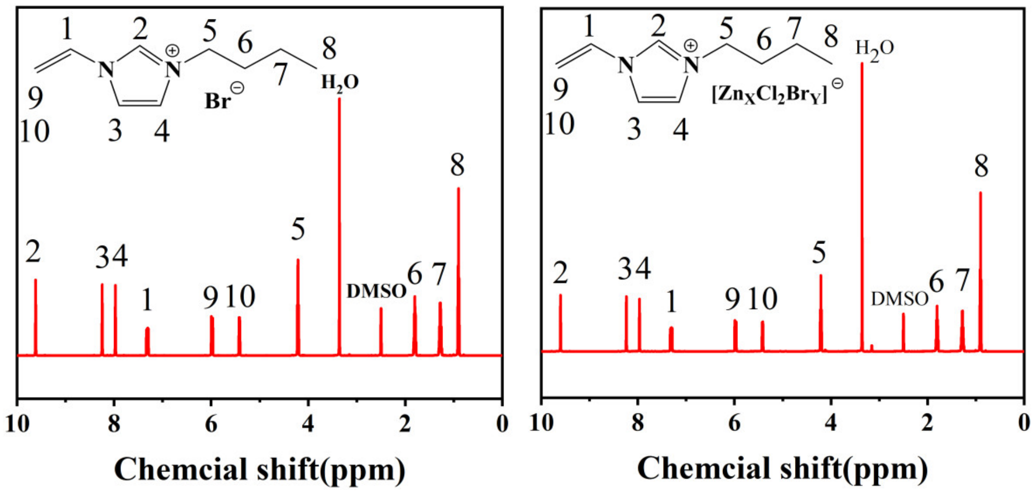

Figure S5. ^{1}H nuclear magnetic resonance (NMR) spectra of [Bvim]Br and [Bmim][$Zn_XCl_2Br_Y$]

Table S1 The value of RL_{max} and EAB for various materials in Fig 5(c).

**Sample**	**RL_{max} (dB)**	**EAB (GHz)**	**Reference**
CoFe@ C-CNTs	-25.7	9	[20]
FeNi alloy and nickel ferrite	-26.2	7.76	[21]
Fe_3O_4@MnO_2@Ni–Co/C	-41.2	7.1	[22]
borate ester bonded hydrogels	-48.7	5.6	[23]
porous carbon	-60.76	6	[24]
organogel	-45.9	5.2	[25]
CuCo nanocube/N-doped carbon nanotube	-54.13	4.01	[26]
graphene@ SiC	-47.5	2.8	[27]
SiC	-43	4	[28]
SiCN	-40	4	[29]
ZnO/MXene	-34.31	3.47	[30]
3D-SiC/PCF-II	-29.74	3.62	[31]
porous fluffy spherical SiC	-28.3	4.15	[32]
Ni-Zn ferrites	-20.8	4.4	[33]
CNTs/VO_2/ANF	-14.2	3.7	[34]
IG-[6]	-45.7	8.08	This work
